# Tracheal Stent Migration in a Patient With Tracheomalacia and Tracheoesophageal Fistula: A Rare Case

**DOI:** 10.7759/cureus.34560

**Published:** 2023-02-02

**Authors:** Mahdi Aljawad, Ahmed Albaqshi, Shahbaz Qazi, Raad Madkhali

**Affiliations:** 1 Radiology, National Guard Health Affairs, Riyadh, SAU

**Keywords:** tracheoesophageal fistula, case report, stent migration, tracheomalacia, tracheal stent

## Abstract

Tracheomalacia refers to diffuse or segmental tracheal weakness. Most commonly, tracheomalacia develops after prolonged endotracheal intubation or tracheostomy. Surgical management is warranted in symptomatic patients with severe tracheomalacia. Relief of airway obstruction via stenting often provides immediate improvement in both airflow and symptoms. However, stent placement is associated with significant complications. Here, we present the case of a 71-year-old man who was brought to the emergency department with acute respiratory distress. The patient was known to have tracheomalacia with tracheoesophageal fistula. He had multiple medical comorbidities, including longstanding hypertension, diabetes mellitus, and asthma. The patient had a progressive decline in his level of consciousness and was admitted to the intensive care unit for further management. Despite the maximum ventilatory support, the patient did not achieve an adequate oxygenation level. The patient underwent tracheal stent placement by the interventional radiology team. The insertion was unsuccessful despite three attempts. The tracheal stent had migrated into the upper esophagus on the first and second insertion attempts. Because the patient was unstable to tolerate further attempts, the multidisciplinary team recommended the insertion of an esophageal stent to cover the tracheoesophageal fistula. Despite this, the patient continued to have air leakage with progressive worsening of his respiratory condition as he developed multiorgan failure and died. The management of tracheomalacia in the setting of the tracheoesophageal fistula may pose several challenges. The present case highlights an essential complication of stent placement with the stent migrating into the tracheoesophageal fistula, which is an unusual site of migration. A multidisciplinary approach is crucial in the management of difficult cases of tracheomalacia.

## Introduction

Tracheomalacia refers to diffuse or segmental tracheal weakness [1]. The prevalence of tracheomalacia in adults is uncertain due to the lack of studies on the general population. In adults, there are several causes of acquired tracheomalacia. Most commonly, tracheomalacia develops after prolonged endotracheal intubation or tracheostomy which can damage the tracheal cartilage and causes tracheal weakness [1]. Other acquired etiologies include chest trauma, malignancy, chronic compression, and relapsing polychondritis. Tracheomalacia may be asymptomatic, especially if the airway narrowing is mild [1,2]. However, clinical symptoms or signs often develop as the airway narrowing progresses or in certain clinical conditions, such as respiratory tract infections [2]. Therapy is warranted in symptomatic patients with severe tracheomalacia. Relief of airway obstruction via stenting often provides immediate improvement in both airflow and symptoms [3]. Here, we present the case of an elderly patient with tracheomalacia and tracheoesophageal fistula who had unusual complications of stent migration into the tracheoesophageal fistula.

This case was previously presented as an abstract at the 2022 Pan Arab Interventional Radiology Society (PAIRS) Annual Congress on May 11-14, 2022.

## Case presentation

A 71-year-old man was brought to the emergency department with acute respiratory distress. The patient had multiple medical comorbidities, including longstanding hypertension, diabetes mellitus, and asthma. He was known to have an acquired tracheomalacia with a tracheoesophageal fistula of idiopathic etiology.

The patient had a progressive decline in his level of consciousness because of the worsening of his respiratory condition. The patient was admitted to the intensive care unit for further management. He had a Glasgow Coma Scale score of 4/15 with no sedation. He had an elevated creatinine level (166 μmol/L) with an estimated glomerular filtration rate of 38 mL/minute in keeping with acute renal failure for which he underwent a hemodialysis session. The patient underwent a computed tomography scan of the thorax which revealed an increase in the size of a previously known loculated fluid collection in the right lung and bilateral pleural effusion. Bilateral chest tubes were placed.

Despite the maximum ventilatory support, the patient did not achieve an adequate oxygenation level. The patient underwent tracheal stent placement by the interventional radiology team. The insertion was unsuccessful despite three attempts.

**Figure 1:**
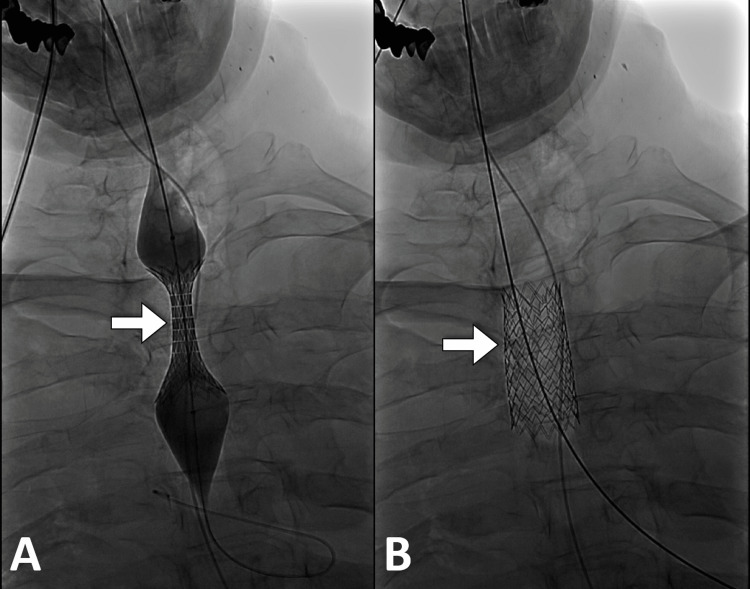
Fluoroscopic images show the insertion of the tracheal stent (arrows) across the tracheoesophageal fistula during (A) and after completion of the insertion (B).

On the first attempt, an oral approach was adopted and a guide wire was secured in the right bronchus. A 24 mm × 58 mm stent was placed to be across the second and third ribs (Figure 1). The patient had a sudden deterioration in oxygen saturation and endotracheal intubation was done. Further examination by bronchoscopy revealed the stent in the upper esophagus.

**Figure 2:**
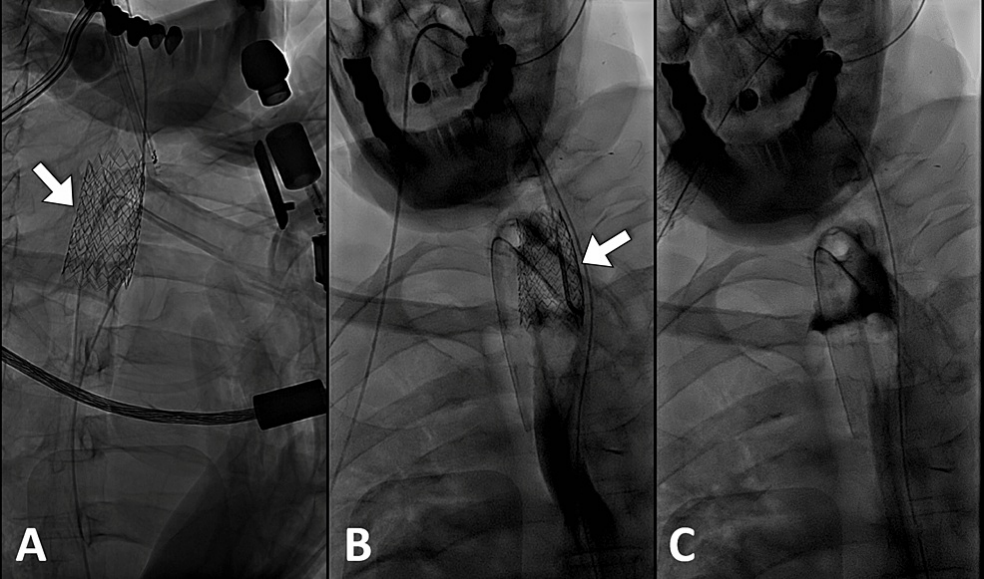
Fluoroscopic images show the reinsertion of the tracheostomy and part of the tracheal stent (arrow) in malposition (A). The position of the tracheal stent (arrow) in the esophagus was confirmed by the upper gastrointestinal study (B). Retained contrast is seen after the removal of the stent (C).

On the second attempt, the stent had migrated to the esophagus through the pre-existing tracheoesophageal fistula causing severe deterioration and the procedure was aborted (Figure 2).

On the last attempt, the stent migrated to the right bronchus, and an attempt to retrieve the stent was made as the patient was unstable for further interventions by the interventional radiology team. Later, the stent was removed endoscopically.

The multidisciplinary team recommended the insertion of an esophageal stent to cover the tracheoesophageal fistula. Under ultrasound aseptic measures and with the use of oral contrast, a guide wire was secured in the jejunum, followed by placement of a 24 mm × 14 cm esophageal stent in the distal esophagus and 28 mm × 20 cm esophageal stent kept in the proximal esophagus with the application of the overlap technique to cover the fistula.

Despite the insertion of the esophageal stent, the air leak persisted. The family members were informed about the condition of the patient and all available attempted interventions failed to cover the fistula. The family agreed on the decision of a do-not-resuscitate plan. The patient remained ventilator-dependent with multiorgan failure. Ten days after the insertion of the esophageal stent, the patient developed hemodynamic instability and asystole (pulseless electrical activity) and died.

## Discussion

We presented a case of tracheal stent migration into the esophagus in a patient with tracheomalacia and tracheoesophageal fistula, which posed several challenges in the management of tracheomalacia. The management of patients with tracheomalacia is ideally done by a multidisciplinary team with input from multiple specialists. The conservative approach can be adopted in asymptomatic adult patients with tracheomalacia [1]. There is a wide variation in the reported estimates of the prevalence of tracheomalacia in the general population. Much of this variation is related to the lack of an exact definition of the degree of dynamic airway compressibility that is significant to cause pathology. However, it should be noted that patients with chronic bronchitis are more than 20 times more common to have tracheomalacia compared to the general population [4].

In patients with severe symptoms, acquired tracheomalacia is likely to need surgical intervention, particularly if tracheomalacia is associated with tracheoesophageal fistula. However, elderly patients with multiple comorbidities may not be candidates for surgical interventions. Hence, stenting remains an alternative in patients with severe symptoms.

Stents are broadly classified into two groups, including plastic (e.g., silicone) and metallic stents (e.g., stainless steel). Several complications of tracheal stents have been described. The most frequent complications are associated with human error in placement, sizing, and overextended placement [5]. The most frequent complications include stent migration, stent fracture, patient intolerance, and the formation of granulation tissue around the stent [6]. In the present case, the management of tracheomalacia via stenting was unsuccessful despite several attempts. It is reported that metallic stents are less likely to migrate compared to plastic stents. Further, migration of stents is more common with stents of shorter lengths. The presence of tracheoesophageal fistula in the current case was an additional risk factor for stent migration. Other reported contributing factors in the literature include the self-expanding nature of the stent, short stent lengths, dynamic size of airways, and location of the stent at the origin of the right mainstem [7]. Stent migration is remedied by endoscopic repositioning or removal and replacement with a more suitable stent. In the present case, all the available options were attempted with no success. While this complication may not be unavoidable in all cases, the risk of stent migration can be decreased by having relatively longer stents.

## Conclusions

The management of tracheomalacia in the setting of the tracheoesophageal fistula may pose several challenges. The present case highlighted an essential complication of stent placement with the stent having migrated into the tracheoesophageal fistula, which is an unusual site of migration. A multidisciplinary approach is crucial in the management of difficult cases of tracheomalacia.
